# *Propionibacterium acnes*: A Treatable Cause of Constrictive Pericarditis

**DOI:** 10.1155/2015/193272

**Published:** 2015-06-17

**Authors:** Daniel Cruz, Haitham Ahmed, Yousuf Gandapur, M. Roselle Abraham

**Affiliations:** ^1^Osler Medical Service, Johns Hopkins School of Medicine, Baltimore, MD 21287, USA; ^2^Division of Cardiology, Department of Medicine, Johns Hopkins Hospital, Baltimore, MD 21287, USA; ^3^Department of Medicine, Good Samaritan Hospital, Baltimore, MD 21239, USA

## Abstract

In this case report we share a case of infective Pericarditis caused by *Propionibacterium acnes* (*P. acnes*) in an immune-competent, nonsurgical patient. This case and review will illustrate the importance of considering *P. acnes* as a cause of idiopathic pericardial effusion and effusive constrictive disease. The patient was a 61-year-old male with history of osteoarthritis of the knee. He received an intra-articular steroid injection in July 2013. Two months later, he presented with atrial fibrillation and heart failure. He was found to have pericardial and bilateral pleural effusions which grew *P. acnes*. This organism was initially considered to be contaminant; however, as *P. acnes* was isolated from both pleural and pericardial fluids, he was started on oral amoxicillin. He was noted to have recurrence of effusions within 2 weeks with evidence of constrictive physiology by echocardiography. Treatment was subsequently changed to intravenous Penicillin G with marked symptomatic improvement, resolution of pericardial/pleural effusions, and no echocardiographic evidence of constrictive pericarditis at 10 weeks follow-up. Pursuit and treatment of *P. acnes* could lead to prevention of constrictive pericarditis. We believe that further studies are needed to assess prevalence of *P. acnes* and response to intravenous Penicillin G in patients presenting with effusive constrictive disease.

## 1. Case Presentation

**Figure 1:**
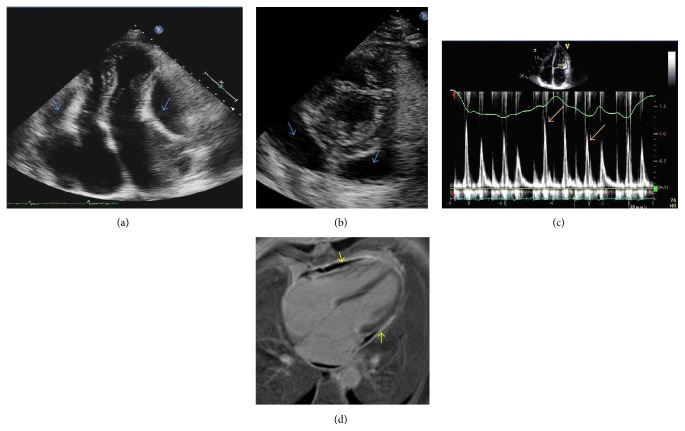
(a), (b) Transthoracic echocardiogram (apical (a) and parasternal short-axis (b)) before pericardiocentesis: two large, loculated pericardial effusions (blue arrows) are noted with significant indentations of the right and left ventricular free wall. (c) Pulsed-wave Doppler interrogation of mitral valve inflow on transthoracic echocardiogram: evidence of significant (>25%) reduction in mitral valve E velocity (orange arrows) consistent with constrictive physiology after pericardiocentesis. (d) Cardiac magnetic resonance imaging following gadolinium administration revealed diffuse pericardial thickening and delayed enhancement (yellow arrows) after pericardiocentesis.

A 61-year-old immune-competent male with history of coronary artery disease preserved ejection fraction, and osteoarthritis of the knee was admitted with a 5-month history of increasing dyspnea, cough, lower extremity edema, and persistent atrial fibrillation. He was diagnosed with bilateral pleural effusions and loculated pericardial effusions (Figures 1(a) and 1(b)).

Pericardiocentesis and bilateral thoracentesis were performed: pericardial and pleural fluid revealed lymphocytic predominance (white blood cell count was 302 with 245 lymphocytes). Pleural fluid was transudate (pH was 7.5, LDH ratio was 0.35, and protein ratio was 0.3). Blood cultures were negative for* P. acnes* and peripheral blood smear showed rare atypical lymphocytes. Hence, the patient underwent a whole body ^18^FDG-PET scan to rule out malignancy. Quantiferon Gold (to rule out tuberculosis) and rheumatologic workup were negative.* P. acnes* was considered contaminant, but since it was isolated from both pleural and pericardial fluids, he was started on oral amoxicillin 1000 mg three times daily, with plans for a 21-day course.

**Figure 2:**
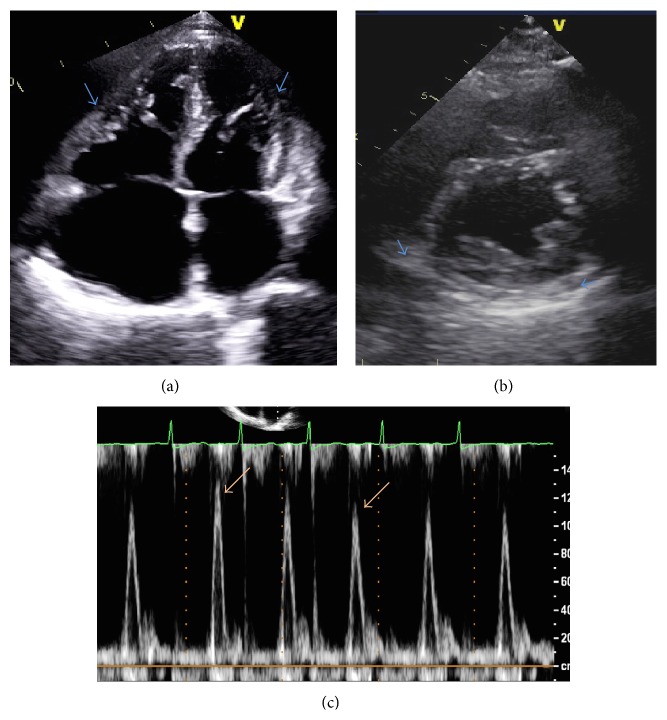
(a), (b) Transthoracic echocardiogram (apical (a) and parasternal short-axis (b)) showing resolution of pericardial effusion (blue arrows) after 10 weeks of IV Penicillin. (c) Pulsed-wave Doppler interrogation of mitral valve inflow on transthoracic echocardiogram: normal variation of mitral valve E velocity (orange arrows), suggesting normalization of pericardial pressure.

The patient was readmitted 2 weeks later with recurrent pericardial and pleural effusions and New York Heart Association (NYHA) class III symptoms. On exam, he did not have evidence of pulsus paradoxus. Jugular venous pressure was 7 cm and systolic blood pressure was in the 110–130 range. He underwent repeat pericardiocentesis and thoracentesis for relief of symptoms. Cultures of pericardial and pleural fluid were negative for* P. acnes* on oral amoxicillin therapy. Echocardiography after pericardiocentesis revealed evidence of constrictive physiology (Figure 1(c)) and MRI demonstrated pericardial thickening with delayed enhancement (following administration of gadolinium), suggesting the presence of active inflammation and/or fibrosis (Figure 1(d)). Intravenous Penicillin G (3 million units every 4 hours) and colchicine 0.6 mg twice daily were initiated for treatment of presumed* P. acnes* pericardial/pleural effusions, with significant improvement in symptoms within a few days. He received a six-week course of intravenous Penicillin G and was subsequently switched to oral doxycycline 100 mg twice daily for 6 weeks. The patient's exercise capacity progressively improved to the point that he was able to participate in an international competitive sporting event several months following discharge. On follow-up, the patient had no recurrence of effusions (Figures 2(a) and 2(b)). Repeat echocardiography performed 10 weeks after initiation of intravenous antibiotics confirmed resolution of constrictive physiology (Figure 2(c)).

## 2. Discussion

*Propionibacterium acnes* (*P. acnes*) is a skin commensal that is recognized as an opportunistic pathogen which has been linked to postoperative and device-related infections, prostatitis, sarcoidosis, and sciatica [1]. Constrictive pericarditis caused by* P. acnes* has been reported most frequently in men, in the setting of cardiac surgery and immunosuppression [2], and requires surgical intervention (pericardial stripping) in a large proportion of patients [3]. Inflammatory cell infiltration and fibrosis confirm that, despite minimal virulence,* P. acnes* has an immune-stimulatory effect on the mononuclear phagocyte system [4].* P. acnes* stimulates production of inflammatory mediators such as metalloproteinases and tumor necrosis factor alpha by macrophages [4], which could lead to exuberant inflammation of the pericardium, pericardial effusions, and constrictive physiology.

Our case reveals that investigation and timely treatment of* P. acnes* could lead to prevention of chronic constrictive pericarditis. However, most standard cultures will not isolate* P. acnes* as it typically takes at least seven days to grow out in culture. Thus, although it is known for its ubiquity, it is a relatively rare result in culture when compared to other well-known pathogens. Based on this result, we recommend ruling out* P. acnes* infection in all patients with pericardial effusions who undergo pericardiocentesis and treatment with a prolonged course of intravenous Penicillin G if cultures reveal* P. acnes*. Prospective studies are needed to examine the prevalence of* P. acnes* infection in patients presenting with pericardial effusions and constrictive physiology (effusive constrictive disease), which would otherwise have been classified as idiopathic.
